# Supplementary material for: An interphase actin wave promotes mitochondrial content mixing and organelle homeostasis
Source: Nat Commun. 2024 May 7;15:3793. doi: 10.1038/s41467-024-48189-1 (PMC11076292; doi:10.1038/s41467-024-48189-1)
Supplement: Supplementary file 1 — Supplementary Information [file 41467_2024_48189_MOESM1_ESM.pdf]

Supplementary Information

An interphase actin wave promotes mitochondrial content mixing and organelle homeostasis

Coscia et al.

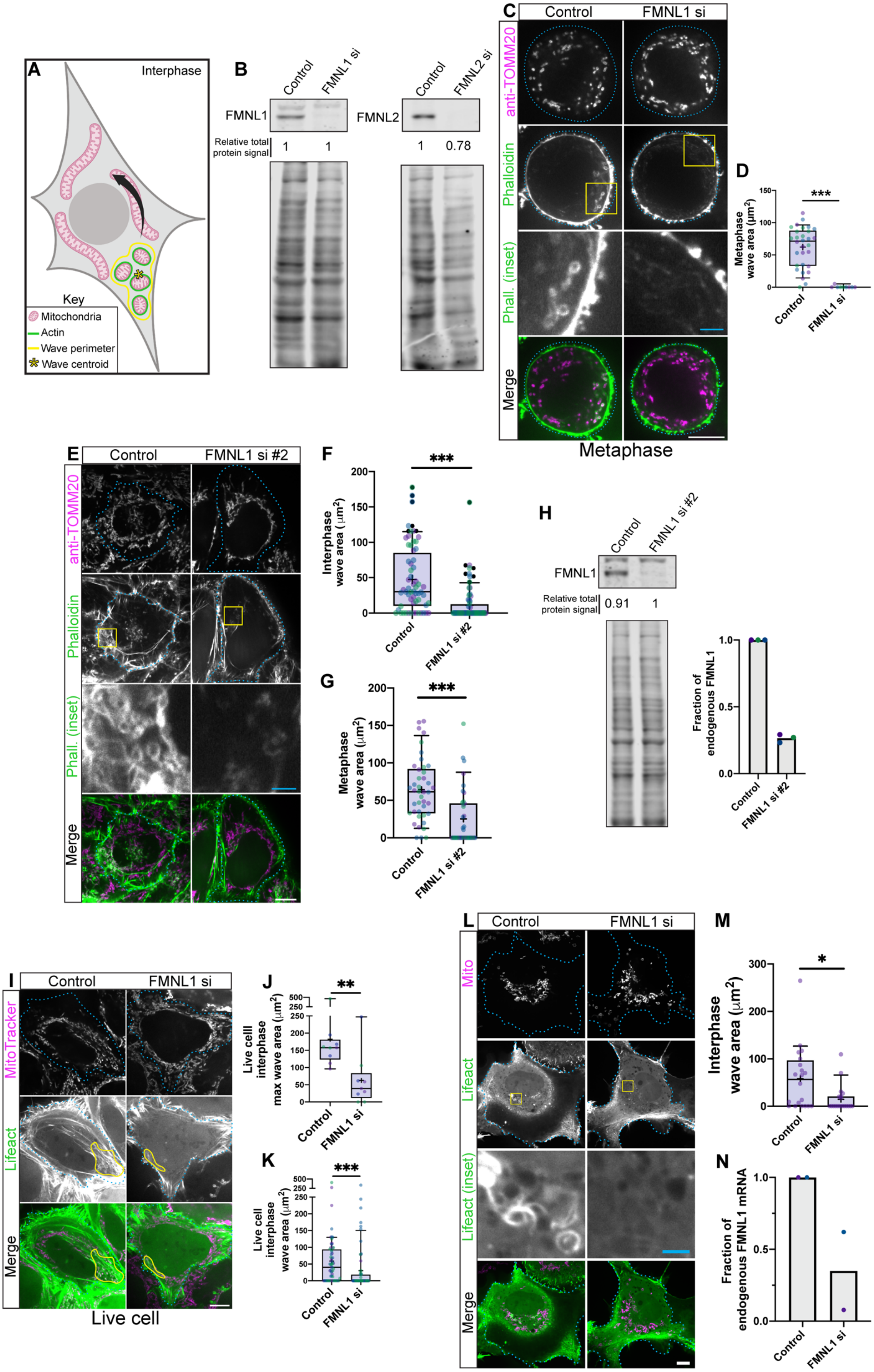
A
Interphase
Key
Mitochondria
Actin
Wave perimeter
Wave centroid
B
Control
FMNL1 si
FMNL1
Relative total protein signal
1
1
Control
FMNL2 si
FMNL2
1
0.78
C
Control
FMNL1 si
anti-TOMM20
Phalloidin
Phall. (inset)
Merge
Metaphase
D
***
Metaphase wave area (µm²)
150
100
50
0
Control
FMNL1 si
E
Control
FMNL1 si #2
anti-TOMM20
Phalloidin
Phall. (inset)
Merge
F
***
Interphase wave area (µm²)
200
150
100
50
0
Control
FMNL1 si #2
G
***
Metaphase wave area (µm²)
200
150
100
50
0
Control
FMNL1 si #2
H
Control
FMNL1 si #2
FMNL1
Relative total protein signal
0.91
1
Fraction of endogenous FMNL1
1.0
0.5
0.0
Control
FMNL1 si #2
I
Control
FMNL1 si
MitoTracker
Lifeact
Merge
Live cell
J
**
Live cell interphase max wave area (µm²)
500
250
250
200
150
100
50
0
Control
FMNL1 si
K
***
Live cell interphase wave area (µm²)
500
250
250
200
150
100
50
0
Control
FMNL1 si
L
Control
FMNL1 si
Mito
Lifeact
Lifeact (inset)
Merge
M
*
Interphase wave area (µm²)
300
200
100
0
Control
FMNL1 si
N
Fraction of endogenous FMNL1 mRNA
1.0
0.5
0.0
Control
FMNL1 si

Fig S1. Requirement of FMNL1 for actin wave is true in different contexts. (a) Cartoon to guide in understanding quantitation of actin wave size and speed. Created with BioRender.com released under a Creative Commons Attribution-NonCommerical-NoDerivs 4.0 International license https://creativecommons.org/licenses/by-nc-nd/4.0/deed.en **(b)** Western blots for FMNL1 and 2 in cells treated with siRNAs to these proteins. **(c)** Representative images of anti-TOMM20 (mitochondrial marker) and phalloidin (F-actin marker) in metaphase cells treated with siRNA to FMNL1. **(d)** Quantitation of wave size. **(e)** Representative images of anti-TOMM20 and phalloidin in interphase cells treated with FMNL1 siRNA #2. **(f)** Quantitation of wave size. **(g)** Same, but for metaphase cells. **(h)** Western blot for experiment examining knock-down efficiency of FMNL1 siRNA #2 with accompanying quantitation. Bars represent means. **(i)** Representative images of MitoTracker Deep Red and Lifeact-GFP in live interphase cells treated with siRNA to FMNL1 where movie frame with largest actin wave is shown. Actin waves are outlined in yellow. Display scaling for mitochondrial channel altered between groups for ease of viewing. **(j)** Quantitation of max wave size in live cells. **(k)** Quantitation of wave size from random timepoint sampling of live cells. **(l)** Representative images of live COS-7 cells in interphase treated with FMNL1 siRNA and expressing Lifeact-GFP and mito-DsRed2. Differential contrasting for mitochondrial channel between groups for ease of viewing. (**m)** Quantitation of wave size from one independent experiment. **(n)** Quantitation of qPCR experiment assessing the knock-down efficiency of our FMNL1 siRNA in COS-7 cells. Bars represent means from two biological replicates. **(all)** Three independent experiments unless otherwise specified. In box plots whiskers represent 10-90th percentile. Center lines indicate medians, and plus signs indicate means. Scale bars are 10μm for whole cell images and 2μm for insets. All statistical tests were two-sided Mann-Whitney tests. Cell boundaries are outlined in cyan. Differently colored points represent different biological replicates. Source data are provided as a Source Data file. *, **, and *** indicate p-values of <0.05, ≤0.005, and ≤0.0005 respectively.

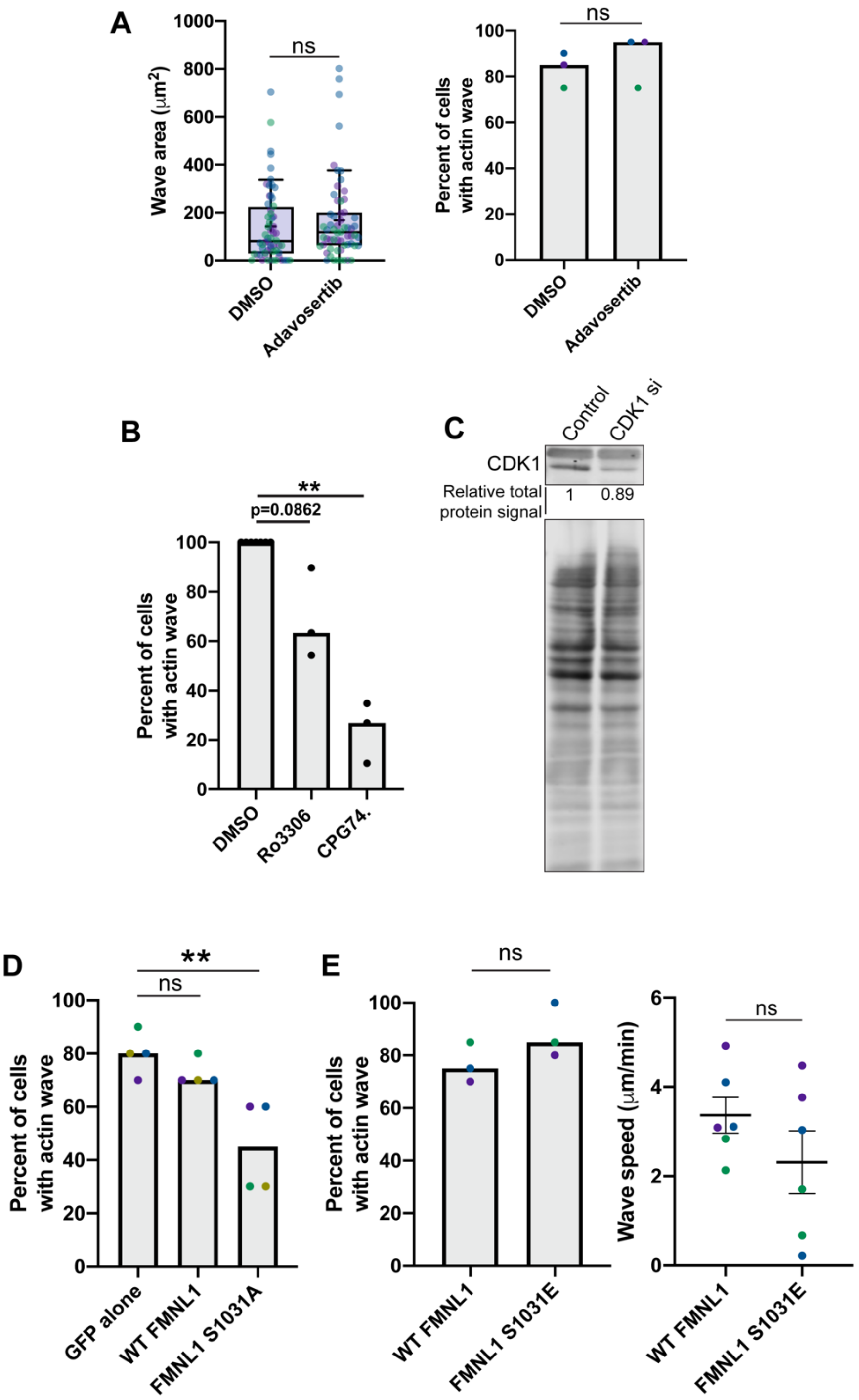

Fig S2. CDK1 acts via FMNL1 to positively regulate the actin wave (a) Quantitation of actin wave area and percent of cells with wave - for cells treated with either vehicle (DMSO) or adavosertib (~4hr, 300nM). A two-sided Mann-Whitney test was used for both graphs. For first graph, whiskers represents 10-90th percentile, center lines indicate medians, and plus signs indicate means. For second graph, bars represent medians. **(b)** Quantitation of percent of cells with wave for cells treated with CDK1 inhibitors. A Kruskal-Wallis test with Dunn's multiple comparisons was run. Bars represent medians. **(c)** Western blot for experiment investigating efficiency of CDK1 siRNA. **(d)** Quantitation of percent of cells with actin wave between cells overexpressing GFP, WT GFP-FMNL1, and FMNL1 S1301A. A Kruskal-Wallis test with Dunn's multiple comparisons was utilized. Bars represent medians. **(e)** Quantitation of percent of cells with a wave and wave speed for cells overexpressing WT FMNL1 and FMNL1 S1031E, with two-sided Mann Whitney and Student's T tests run, respectively. Bars represent medians and means with SEM, respectively. **(all)** 3 or more independent experiments. Different colored points indicate different biological replicates. Source data are provided as a Source Data file. *, **, and *** indicate p-values of <0.05, ≤0.005, and ≤0.0005 respectively.

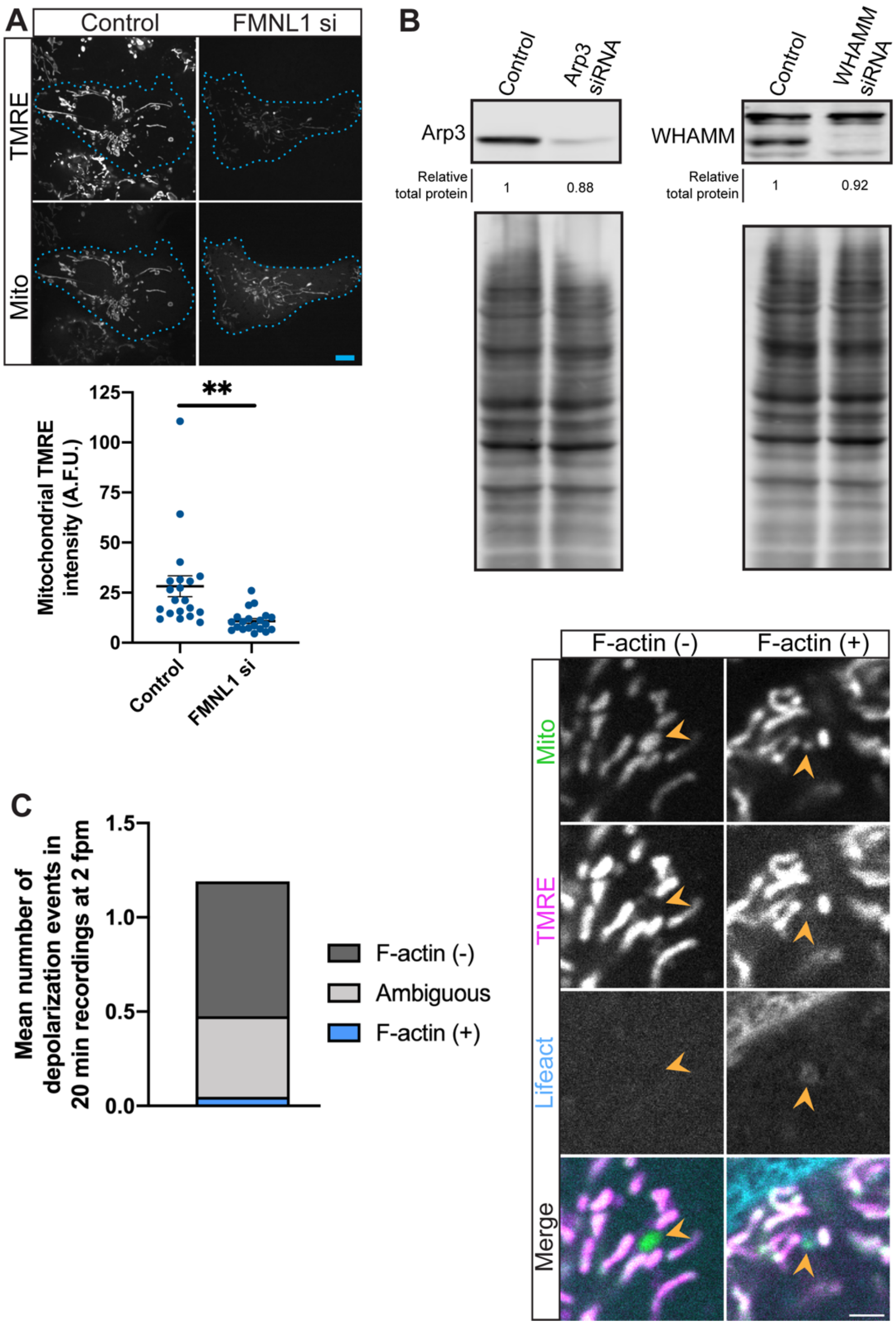

Fig S3. F-actin-positive mitochondrial depolarization event frequency in cultured HeLa cells. (a) Representative images of TMRE and mito-sBFP2 in control and FMNL1 knock-down COS-7 cells. Between conditions mitochondrial channel isn't equally display scaled for ease of viewing. Scale bar is 10μm. Images are accompanied by quantitation where a two-sided Student's T test was used. Lines indicate means and error bars are SEM. One biological replicate. **(b)** Western blots for experiment examining knock-down efficiencies of ARP3 and WHAMM siRNAs. **(c)** Images of mito-SNAP, TMRE, and Lifeact in live interphase cells where an actin negative and actin positive mitochondrial depolarization event is shown. These events are indicated by orange arrows and the scale bar is 2μm. Images are accompanied by quantitation of number of depolarization events in 20 minute recordings at 2 frames per minute. Total n = 21 cells over 3 independent experiments. Source data are provided as a Source Data file. *, **, and *** indicate p-values of <0.05, ≤0.005, and ≤0.0005 respectively.

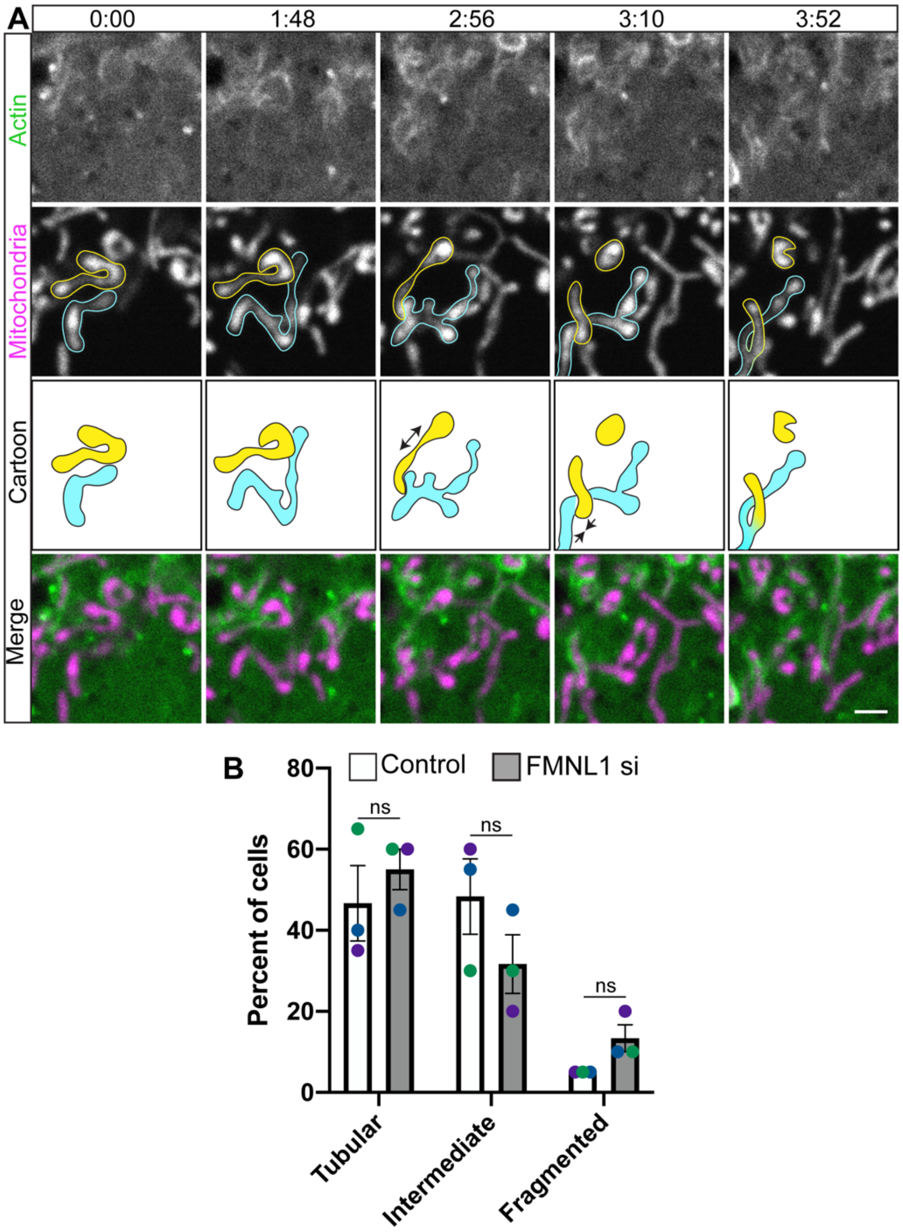

Fig S4. FMNL1 depletion does not alter gross mitochondrial morphology. (a) Representative images of interphase COS-7 cell expressing Lifeact-GFP and mito-DsRed2, where heterofusion as a result of the actin wave is exhibited. Scale bar is 2µm. One independent experiment. **(b)** Quantitation of mitochondrial morphology in control cells and FMNL1 knock-down live HeLa cells expressing Lifeact-GFP and stained with MitoTracker Deep Red FM. 3 biological replicates represented by different colors. Means are depicted with SEM, where a two-way ANOVA with multiple comparisons was used for statistical analysis. Source data are provided as a Source Data file.

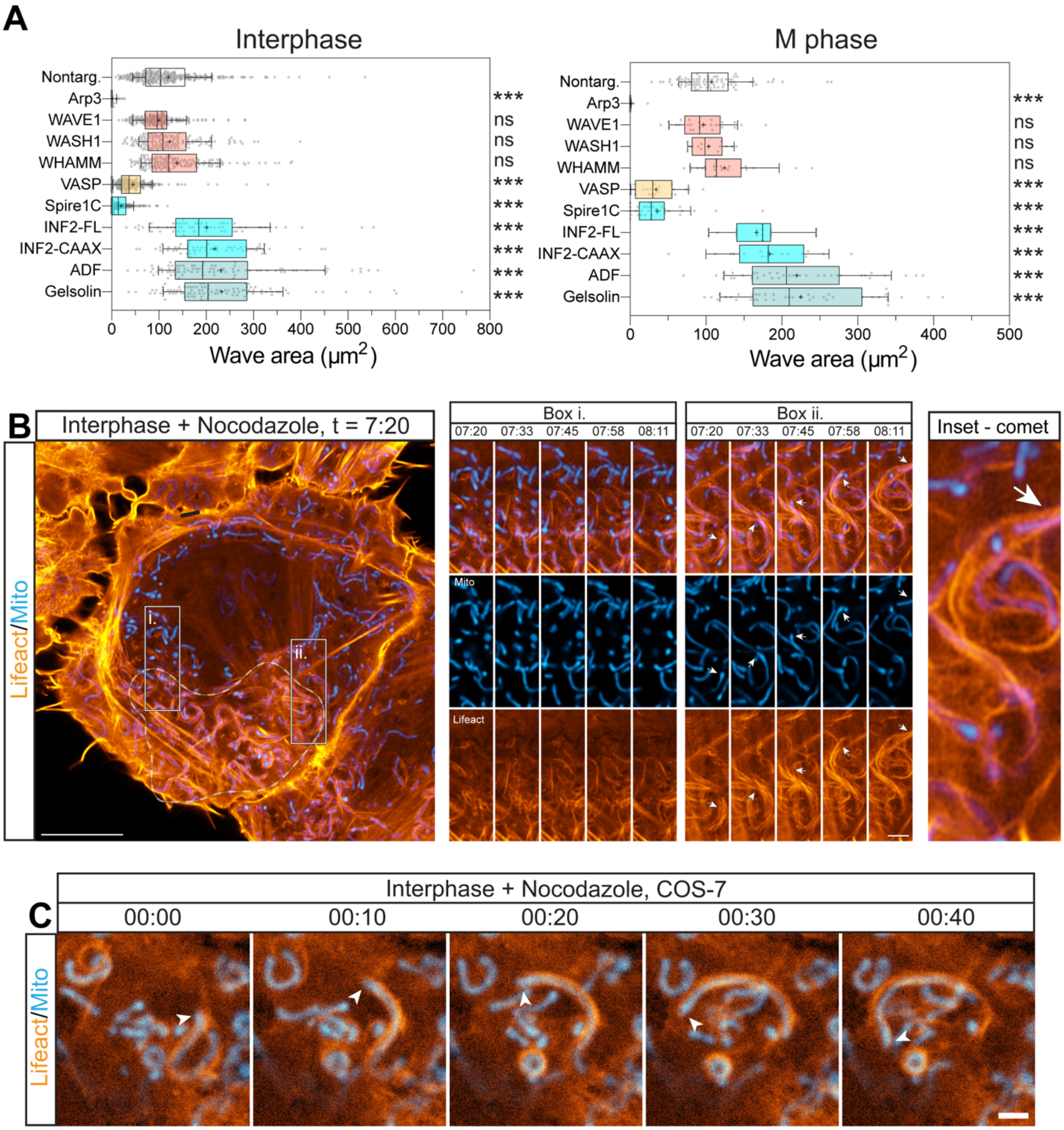

Fig S5. The presence or absence of microtubules dictates actin wave-mediated mitochondrial dynamics in different contexts (a) Cross-sectional area of interphase or m-phase actin waves after depletion of the indicated actin binding proteins. 10-90% shown, where center lines indicate medians and plus signs indicate means. A Kruskal-Wallis test with Dunn's multiple comparisons was run. Source data from Moore et al., 2021. ≥3 independent experiments for most conditions, for some, 2 biological replicates including those with effects: interphase ADF and metaphase Arp3. Red is NPFs, light blue is F-actin nucleators/elongators, and dark blue is F-actin depolymerizers. **(b)** Airyscan. Actin comet tail (Lifeact-eGFP, orange) associated with a mitochondrion (mito-DsRed2, blue) in a 1h 10μM nocodazole treated interphase HeLa cell. Cell also expressing halo-Sec61B and labeled with Janelia Fluor 635 (not shown). Scale bar is 10μm left, 2μm right**. (c)** Example image of actin wave-associated mitochondrial comet tail in COS-7 cell expressing Lifeact-GFP and mito-DsRed2 treated with 25μM nocodazole for 1hr. Scale bar is 2μm. Source data are provided as a Source Data file. *, **, and *** indicate p-values of <0.05, ≤0.005, and ≤0.0005 respectively.
