# Supplementary material for: An interphase actin wave promotes mitochondrial content mixing and organelle homeostasis
Source: Nat Commun. 2024 May 7;15:3793. doi: 10.1038/s41467-024-48189-1 (PMC11076292; doi:10.1038/s41467-024-48189-1)
Supplement: Supplementary file 3 — Description of Additional Supplementary Files [file 41467_2024_48189_MOESM3_ESM.pdf]

Description of Additional Supplementary Files

File name: Supplementary Movie 1
Description: Interphase actin wave samples mitochondrial subpopulations promoting their fragmentation. Video of interphase HeLa cell where actin is marked with Lifeact-GFP (green) and mitochondria are marked with mito-DsRed2 (magenta). Arrow indicates the position of the actin wave. Time = min:sec.

File name: Supplementary Movie 2
Description: CDK1 activation increases speed of interphase actin wave. Video of HeLa cells in interphase where actin is marked with Lifeact-GFP, after addition of DMSO or the WEE1 inhibitor Adavosertib (300nM for ~4hrs). Arrows indicate the positions of the actin waves. Time = min:sec. Display scaling between the two conditions is not equal for ease of viewing.

File name: Supplementary Movie 3
Description: GCaMP6s signal is not correlated with the interphase actin wave. Video of interphase HeLa cell expressing the calcium indicator GCaMP6s and the actin marker Lifeact-miRFP. Arrow indicates the position of the actin wave. Time = min:sec.

File name: Supplementary Movie 4
Description: The actin wave persists after mitochondrial depolarization. Video of interphase HeLa cells where actin is marked with Lifeact-GFP, both without drug addition and after 20μM CCCP for >30 minutes. Arrows indicate the positions of the actin waves. Time = min:sec.

File name: Supplementary Movie 5
Description: Interphase actin wave-associated mitochondrial actin comet tails form after microtubule depolymerization. Video of interphase HeLa cell treated with 10μM nocodazole for 1hr, where actin is marked with Lifeact-GFP and mitochondria are marked with mito-DsRed2. Arrow indicates the leading edge of an actin wave-associated mitochondria with a trailing actin comet tail. Time = min:sec.
