# Supplementary material for: An interphase actin wave promotes mitochondrial content mixing and organelle homeostasis
Source: Nat Commun. 2024 May 7;15:3793. doi: 10.1038/s41467-024-48189-1 (PMC11076292; doi:10.1038/s41467-024-48189-1)
Supplement: Supplementary file 9 — Reporting Summary [file 41467_2024_48189_MOESM9_ESM.pdf]

nature portfolio

Corresponding author(s): Erika L.F. Holzbaur

Last updated by author(s): Feb 20, 2024

Reporting Summary

Nature Portfolio wishes to improve the reproducibility of the work that we publish. This form provides structure for consistency and transparency in reporting. For further information on Nature Portfolio policies, see our Editorial Policies and the Editorial Policy Checklist.

Statistics

For all statistical analyses, confirm that the following items are present in the figure legend, table legend, main text, or Methods section.

n/a	Confirmed	
☐	☒	The exact sample size (*n*) for each experimental group/condition, given as a discrete number and unit of measurement
☐	☒	A statement on whether measurements were taken from distinct samples or whether the same sample was measured repeatedly
☐	☒	The statistical test(s) used AND whether they are one- or two-sided<br>*Only common tests should be described solely by name; describe more complex techniques in the Methods section.*
☒	☐	A description of all covariates tested
☐	☒	A description of any assumptions or corrections, such as tests of normality and adjustment for multiple comparisons
☐	☒	A full description of the statistical parameters including central tendency (e.g. means) or other basic estimates (e.g. regression coefficient) AND variation (e.g. standard deviation) or associated estimates of uncertainty (e.g. confidence intervals)
☐	☒	For null hypothesis testing, the test statistic (e.g. *F*, *t*, *r*) with confidence intervals, effect sizes, degrees of freedom and *P* value noted<br>*Give P values as exact values whenever suitable.*
☒	☐	For Bayesian analysis, information on the choice of priors and Markov chain Monte Carlo settings
☒	☐	For hierarchical and complex designs, identification of the appropriate level for tests and full reporting of outcomes
☒	☐	Estimates of effect sizes (e.g. Cohen's *d*, Pearson's *r*), indicating how they were calculated

Our web collection on statistics for biologists contains articles on many of the points above.

Software and code

Policy information about availability of computer code

Data collection	No software was used in data collection.
Data analysis	Raw MS files were processed using MaxQuant and visualized in Scaffold (Proteome Software, Portland Oregon)

For manuscripts utilizing custom algorithms or software that are central to the research but not yet described in published literature, software must be made available to editors and reviewers. We strongly encourage code deposition in a community repository (e.g. GitHub). See the Nature Portfolio guidelines for submitting code & software for further information.

Data

Policy information about availability of data

All manuscripts must include a data availability statement. This statement should provide the following information, where applicable:

- Accession codes, unique identifiers, or web links for publicly available datasets
- A description of any restrictions on data availability
- For clinical datasets or third party data, please ensure that the statement adheres to our policy

Source data are provided with this paper. Mass spectrometry data have been deposited to the ProteomeXchange Consortium via the PRIDE partner repository with the dataset identified PXD050085. The raw imaging data are available under restricted access for reason of large file sizes, access can be obtained by requesting from the corresponding author.

Research involving human participants, their data, or biological material

Policy information about studies with human participants or human data. See also policy information about sex, gender (identity/presentation), and sexual orientation and race, ethnicity and racism.

Reporting on sex and gender	N/A
Reporting on race, ethnicity, or other socially relevant groupings	N/A
Population characteristics	N/A
Recruitment	N/A
Ethics oversight	N/A

Note that full information on the approval of the study protocol must also be provided in the manuscript.

Field-specific reporting

Please select the one below that is the best fit for your research. If you are not sure, read the appropriate sections before making your selection.

☒ Life sciences ☐ Behavioural & social sciences ☐ Ecological, evolutionary & environmental sciences

For a reference copy of the document with all sections, see nature.com/documents/nr-reporting-summary-flat.pdf

Life sciences study design

All studies must disclose on these points even when the disclosure is negative.

Sample size	For experiments with a sample size less than or equal to 17, we performed a power analysis and increased sample size as needed, except for experiments evaluating percent wave-positive cells and Fig. 3B,C, & E, where due to the time-intensive imaging and data analysis involved, additional biological replicates beyond a minimum of 3 could not be performed. However for these experiments, statistically significant changes were determined using superplot analysis according to Lord et al., JCB 2020. Other experiments, measuring actin wave size, featuring higher sample sizes, were analyzed without power analysis consistent with previous work in the field (Moore et al., Nature 2021).
Data exclusions	In Fig S1D cells with clearly misaligned chromosomes were excluded, as noted in the original and revised manuscripts. In Fig 3B some cells which appeared unhealthy were excluded. Exclusion criteria were not pre-established.
Replication	All experimental replicates are reported. Within some data sets, there was trial-to-trial variability, but analysis of the complete data sets were statistically significant as noted. For the vast majority of data generated for this study, unless otherwise noted, at least 3 biological replicates were performed per experiment.
Randomization	Not relevant to study, as we use a cell culture model. Experimental and control groups were directly taken from the same pool of cells and thus were identical prior to treatments.
Blinding	Experiments were performed and analyzed in a blinded manner except for: in Fig S1C & Fig2H where effect size was very large and trend therefore obvious, Fig 4D where there was clearly no effect, and Fig S6A where the data was produced as part of a larger screen, as noted in the text.

Reporting for specific materials, systems and methods

We require information from authors about some types of materials, experimental systems and methods used in many studies. Here, indicate whether each material, system or method listed is relevant to your study. If you are not sure if a list item applies to your research, read the appropriate section before selecting a response.

Materials & experimental systems

n/a	Involved in the study
☐	☒ Antibodies
☐	☒ Eukaryotic cell lines
☒	☐ Palaeontology and archaeology
☒	☐ Animals and other organisms
☒	☐ Clinical data
☒	☐ Dual use research of concern
☒	☐ Plants

Methods

n/a	Involved in the study
☒	☐ ChIP-seq
☒	☐ Flow cytometry
☒	☐ MRI-based neuroimaging

Antibodies

Antibodies used

For western blots and immunofluorescence the following primary antibodies were used: TOMM20 (Santa Cruz, sc-17764), FMNL1 (Abcam, ab97456), FMNL2 (Santa Cruz, sc-390298), GFP (abcam, ab1218), GFP (Aves, 1020), phospho-CDK1 substrate consensus sequence (CST, 2325), CDK1 (abcam, ab131450), ARP3 (Proteintech, 13822-1-AP), WHAMM (abcam, ab122572). Coupled to these primary antibodies were the following secondaries: anti-mouse Alexa Fluor 647 (ThermoFisher, A32728), anti-mouse AlexaFluor 405 (ThermoFisher, A31553), anti-Rabbit IRDye 800CW (LI-COR, 926-32213), and anti-Mouse IRDye 800CW (LI-COR, 626-32212).

Validation

For TOMM20, the manufacturer has performed HeLa cell knock-down validation via Western blot. For FMNL1, we have carried out HeLa cell knock-down validation via Western blot here (Fig 1E & Fig S1B). For FMNL2, we have performed HeLa cell knock-down validation via Western blot here (Fig 1E & Fig S1B). For both GFP antibodies the manufacturer has produced validation via images of western blots and immunofluorescence. The abcam GFP antibody is further validated in the literature (for example, Havkin-Solomon et al Nucleic Acids Research 2023), as well as the Aves GFP antibody (for example Zhang et al Nature Communications 2023 PMID:38081810). For the phospho-CDK1 substrate consensus sequence antibody supporting evidence for its specificity can be found in Jones et al., 2018 Journal of Cell Biology. For CDK1 we have performed knock-down validation via western (Fig 2J & S2C). For ARP3 and WHAMM we have performed knock-down validation via western (Fig3F & S3A).

Eukaryotic cell lines

Policy information about cell lines and Sex and Gender in Research

Cell line source(s)

HeLa-M cells, originally from female donor, were obtained from A. Peden (Cambridge Institute for Medical Research, Cambridge, UK). HeLa-M cells stably expressing Lifeact-GFP and MitoDsRed2 were previously described in Moore et al., 2021. COS-7 cells from ATCC were also utilized.

Authentication

HeLa-M cells (majority of experiments) were authenticated by STR profiling. The vendor of the COS-7 cells authenticates these cells using a PCR assay with species-specific primers, specifically for CO1.

Mycoplasma contamination

Almost all experiments were done in HeLa-M cells and these tested negative for mycoplasma. COS-7 cells tested negative previously (Fenton et al., Nature Communications 2021).

Commonly misidentified lines
(See ICLAC register)

No commonly misidentified cell lines were used in the study.

Plants

Seed stocks

Report on the source of all seed stocks or other plant material used. If applicable, state the seed stock centre and catalogue number. If plant specimens were collected from the field, describe the collection location, date and sampling procedures.

Novel plant genotypes

Describe the methods by which all novel plant genotypes were produced. This includes those generated by transgenic approaches, gene editing, chemical/radiation-based mutagenesis and hybridization. For transgenic lines, describe the transformation method, the number of independent lines analyzed and the generation upon which experiments were performed. For gene-edited lines, describe the editor used, the endogenous sequence targeted for editing, the targeting guide RNA sequence (if applicable) and how the editor was applied.

Authentication

Describe any authentication procedures for each seed stock used or novel genotype generated. Describe any experiments used to assess the effect of a mutation and, where applicable, how potential secondary effects (e.g. second site T-DNA insertions, mosiacism, off-target gene editing) were examined.
